# Characterization of in vivo binding kinetics and non-displaceable binding of [^18^F]SynvesT-1 in the rat brain

**DOI:** 10.1186/s13550-025-01270-2

**Published:** 2025-06-18

**Authors:** Catriona Wimberley, Carlos J. Alcaide-Corral, Timaeus E. F. Morgan, Mark G. Macaskill, Bernadette Andrews, Holly McErlain, Valeria K. Burianova, Andrew Sutherland, Adriana A. S. Tavares

**Affiliations:** 1 School of Physics and Astronomy, University of Edinburgh, Edinburgh, United Kingdom; 2 Edinburgh Imaging, University of Edinburgh, Edinburgh, United Kingdom; 3 Centre for Cardiovascular Science, BHF-University of Edinburgh, Edinburgh, United Kingdom; 4 School of Chemistry, University of Glasgow, Glasgow, United Kingdom

**Keywords:** Rat, [^18^F]SynVesT-1, SV2A, Mass dose, Preclinical PET

## Abstract

**Background:**

The synaptic vesicle glycoprotein 2 A (SV2A) has been identified as a biomarker of interest for neurological pathology. The SV2A specific radiotracer [^18^F]SynVesT-1 has shown good binding characteristics in mouse and human. The aim of this study was to characterize the binding parameters of [^18^F]SynVesT-1 in the rat brain and investigate simplified quantification methods. Twenty-one Positron Emission Tomography (PET) scans were conducted in male Sprague-Dawley rats with a bolus injection of [^18^F]SynVesT-1. Varying concentrations of non-radioactive SynVesT-1 were injected in an increasing mass dose paradigm (*n* = 21 ) with radioactivity in arterial blood recorded throughout. The radiometabolism was characterized in a further group (*n* = 7). The total volume of distribution (*V*_*T*_) was estimated using compartmental modelling and Logan plot and then compared to the standardized uptake value at 30–60 min (*SUV*_*30 − 60*_). Occupancy plots and a Lassen plot were generated.

**Results:**

The pharmacokinetics of [^18^F]SynVesT-1 PET showed rapid brain uptake and increasing doses of SynVesT-1 revealed a robust reduction in radiotracer uptake over all brain regions. The two-tissue compartmental model was most appropriate and the estimated *V*_*T*_ was highly correlated with Logan *V*_*T*_, as was the *SUV*_*30 − 60*_. The *V*_*ND*_ was estimated to be 3.75, which is 12.5% (pons) to 22% (thalamus) of the *V*_*T*_. The estimated upper mass limit required to achieve 5% target occupancy is 0.48 µg/kg.

**Conclusion:**

[^18^F]SynVesT-1 shows good characteristics for imaging the rat brain, however care must be taken to achieve adequate molar activity to avoid mass dose affects (< 5% occupancy). Data showed no suitable reference region for [^18^F]SynVesT-1, however *SUV*_*30 − 60*_ does give an appropriate surrogate for *V*_*T*_.

**Clinical trial number:**

Not applicable.

**Supplementary Information:**

The online version contains supplementary material available at 10.1186/s13550-025-01270-2.

## Introduction

The synaptic vesicle glycoprotein 2 A (SV2A) is a transmembrane protein within synaptic vesicles which exists alongside two other isoforms, SV2B and SV2C. SV2A has expression throughout the brain and the specificity for synapses means it has been identified as an interesting target of biomarker development for many neurological applications.

The epilepsy drug levetiracetam binds to SV2A selectively and has been the basis for development of multiple radiotracers, the first of which, [^11^C]UCB-J, was shown to correlate with ex vivo measures of SV2A in the non-human primate [1]. Since then, a number of other radiotracers for SV2A have been developed, including [^18^F]SynVesT-1, which has favorable binding kinetics [2, 3].

The functions of SV2A are still not fully elucidated but it is thought to have a strong role in neurotransmission among other roles [4]. Studies of SV2A protein using [^18^F]SynvesT-1 PET have shown differences in different pathologies, in rodent [5–12] and in human [13–22]. Additionally, numerous studies have examined SV2A expression in the rat brain, as measured by autoradiography and ex vivo techniques [23] including findings that show SV2A differences between glutamatergic and GABAergic terminals throughout postnatal development [24]. PET studies of SV2A in the rat brain have shown differences in several brain regions in a model of epilepsy [25], Parkinson’s and Huntington’s disease [26, 27]. To date there have been several studies characterizing [^18^F]SynVesT-1 in the mouse [6, 7, 10, 28]; and one study characterizing the PET kinetic behaviour in the rat in vivo [29]. To understand how [^18^F]SynVesT-1 binding to SV2A changes in pathology, it is first important to know the binding characteristics and the response of the target to increasing doses of compound, as this can be instrumental to define best outcome measures and whether reference tissue methods are suitable. To that end, the aim of this study was to conduct a mass dose experiment and estimate the non-displaceable binding (*V*_*ND*_), the upper mass dose limits for rat studies and equivalent for human studies. Additionally, the use of a simplified quantification measure, the standardised uptake value (SUV), as a surrogate for the volume of distribution *V*_*T*_ was investigated.

## Materials and methods

### Radiotracer synthesis

The radiosynthesis of [^18^F]SynVesT-1 was performed as previously described [3, 30] albeit with a precursor enantiomeric purity of 80% for the PET scans and between 80% and 97% for the radiometabolite studies.

### Animals and surgical procedures

All the experiments involving animals were authorized by the local University of Edinburgh Animal Welfare and Ethical Review Committee and in accordance with the Home Office Animals (Scientific Procedures) Act 1986. For the experimental procedures and recording, the ARRIVE guidelines were followed. The animals were housed under standard 12 h light: 12 h dark conditions with food and water available *ad libitum*. Male naïve adult Sprague Dawley rats were used for imaging experiments (*n* = 21, 13.7 ± 2.1 weeks, 483 ± 136 g, mean ± SD) and radiometabolite experiments (*n* = 7, 16.9 ± 4 weeks, 542.9 ± 89 g, mean ± SD).

For imaging experiments, an intravenous line was established in the femoral vein for injection of the radiotracer, and the femoral artery was cannulated to allow automated blood sample collection, as previously described [31]. For radiometabolite experiments, the femoral artery was cannulated for blood sampling and the radiotracer was administered via the tail vein. Surgical cannulation of the femoral vein and artery was performed as described in a previous study [32].

### Metabolite analysis and plasma to whole blood ratio

Arterial blood samples and heart, lung, brain, spleen, liver and kidney tissue samples were collected at 2, 5, 10, 20, 30 and 60 min post-[^18^F]SynVesT-1 injection (58.2 ± 17 MBq, mean SD, *n* = 7), placed on ice and processed immediately. Blood and tissue samples were processed and analyzed as described in a previous study [32]. Briefly, all blood samples were 1 mL each and manually collected from different animals. Following blood and tissue collection, all samples were kept on ice until analyzed. Radioactivity in whole blood and plasma, as well as homogenized tissue samples, was assessed using a well-type γ-counter using a 400–1400 keV window (Perkin Elmer Wizzard2, USA). Plasma samples (400–600 µl) and tissue samples (1000 µl) were processed by acetonitrile denaturation (1:1.4 v/v) and analyzed by high performance liquid chromatography (Ultimate2000, ThermoFisher, UK) on a Luna C18(2) 10 × 250 mm, 10 μm column (Phenomenex, UK) with acetonitrile/water 50/50 and flow rate of 4 mL/min to estimate the parent fraction. The parent fraction curve was fitted with the Watabe model as implemented in PMOD version 4.4 (PMOD Technologies, Switzerland).

### PET/CT acquisition and reconstruction

Healthy rats were anaesthetized using 2% isofluorane gas (IsofloR APIECE, Zoetis, UK) in a 1 L/min, 50/50 oxygen/nitrous oxide mixture. Initially, a computed tomography (CT) image was acquired (nanoPET/CT, Mediso, Hungary) for 5 min and then each animal underwent dynamic PET scanning for two hours following administration of [^18^F]SynVesT-1 (37.2 ± 10.7 MBq). 3 scans were kept for baseline analysis (molar activity = 10.9 ± 2.60 GBq/µmol at end of synthesis). 12 animals had nonradioactive SynVesT-1 diluted in DMSO injected alongside the radioactive tracer (Dose 1 to Dose 4: 0.01, 0.1. 0.5 and 1 mg/kg injected, *n* = 3 per dose, molar activity 59.3 ± 23.65 GBq/µmol at end of synthesis) and one further cohort of animals were scanned with DMSO (vehicle) alone injected alongside the radioactive tracer (*n* = 6, molar activity = 79.0 ± 13.15 GBq/µmol at end of synthesis). Throughout each PET scan, there was arterial blood sampling carried out using the Twilite blood sampling device [31].

CT imaging was conducted using semi-circular full trajectories, maximum field of view, 480 projections, 50 kVp, 300 ms exposure time and 1:4 binning. The CT reconstruction was performed using filtered backprojection and the following parameters: 250 × 250 × 250 μm voxel size, cosine filter, 100% cut-off, and corrections for offset, gain and pixel. Dynamic PET imaging was conducted using the 1:5 scanning mode and packet timestamp list mode with a 50% field of view overlap. All PET studies were reconstructed as 0–2 h post-injection imaging datasets. PET studies were reconstructed using the Mediso iterative Tera-Tomo 3-dimensional reconstruction algorithm, which includes point-spread correction, and the following settings: 4 iterations, 6 subsets, full detector model, normal regularisation, spike filter on, voxel size of 0.2 mm, and 400–600 keV energy window. Dynamic PET framing was performed as follows: 18 × 10 s; 1 × 30 s; 1 × 60 s; 2 × 120 s; 10 × 300 s; and 6 × 600 s. All PET data were corrected for randoms, scatter and attenuation.

### Image processing and kinetic modelling

The percent parent fraction in blood from the radiometabolite study was used as a population-based curve to correct the arterially sampled whole blood curve. There was no whole blood to plasma ratio applied as this ratio was found to be 1 and stable at multiple time points.

Reconstructed PET/CT images were analysed in Anatomist & BrainVISA (v. 5.1.1 https://brainvisa.info/web/index.html). One CT scan from the baseline group was designated the reference and registered to the Schiffer atlas from the PMOD software (PMOD Technologies, Switzerland). To minimize volumetric effects of brain regions in the Schiffer atlas (200 g rats) versus our animals (250–300 g), scaling was used to ensure optimal alignment of reference versus input images. All other CT scans were manually registered with the designated reference CT. The CT to atlas transformation matrices were applied to the PET/CT scan to put them into the same space as the Schiffer atlas. Time activity curves (TACs) were extracted for six brain regions in the Schiffer atlas for each PET scan including: striatum, cortex, hippocampus, cerebellar grey matter, thalamus and pons. The TACs and a radiometabolite corrected arterial input function were used as input for compartmental modelling which was carried out in PMOD version 4.4 (PMOD Technologies, Switzerland). One and two-tissue compartmental models were tested (1TC and 2TC), with a fixed blood volume (vB) of 5%. The Logan plot was also applied to the data for each region of interest and animal using a fixed t* of 34 min which was taken as the average t* from a first pass Logan analysis where it was left floating. The appropriate compartmental model was chosen for the baseline dataset using the Akaike criterion (AIC) and the Logan plot *V*_*T*_ values were compared to the *V*_*T*_ values from the 2TC modelling for the baseline scans. *V*_*T*_ values were excluded where there was a standard error > 50%. The regional *V*_*T*_ values from the Logan plot modelling were used in a Lassen plot to find the *V*_*ND*_ value and the receptor occupancy at each dose level. A regional occupancy plot was generated and fitted with a one-site binding equation for estimation of the affinity, *K*_*d*_ using Prism 10.2.3 (GraphPad, US). The mass dose limits *D*_*5*_ and *D*_*10*_ were estimated from the occupancy plot fit, as per Knyzeliene et al. [33] and to calculate the upper mass dose limit for a rat and a human, the lowest regional D5 mass dose limit was used and multiplied by an average weight for a rat (200 g) and a human (70 kg). The SUV at different time points (20–40 min and 30–60 min) is also compared to the *V*_*T*_ from compartmental modelling and the Logan plot.

### Statistical analysis

Plotting of graphs and statistical analysis were performed with Prism 10.2.3. A mixed effects model with Sidak’s post hoc test (alpha = 0.05) was applied to the mass effect data over the brain regions included. Pearson correlation analyses were performed between variables (*V*_*T*_ from 2TC and Logan analysis) and the bias introduced by using the Logan plot was quantified in a Bland Altman plot.

### Occupancy calculations

The Lassen plot was generated using a linear regression analysis between values from each injected mass level. Occupancy was calculated as per Eq. (1) for generation of an occupancy plot:

1 $$\:100\:\times\:\:\frac{{V}_{S-vehicle}-{V}_{S-dose}}{{V}_{S-vehicle}}$$.

Where the *V*_*S*_*= V*_*T*_-*V*_*ND*_ and *V*_*ND*_ was taken as the global estimate from the Lassen plot. Fitting of the occupancy plot was done with a one and two site specific model (Prism 10.2.3) and stability of the estimated parameters used to select the model. The formula from the model was used to estimate the D5 and D10 (injected mass required for 5% and 10% occupancy respectively) and was also used to calculate the occupancy for the three control experiments.

## Results

### Plasma and rat brain activity

**Fig. 1:**
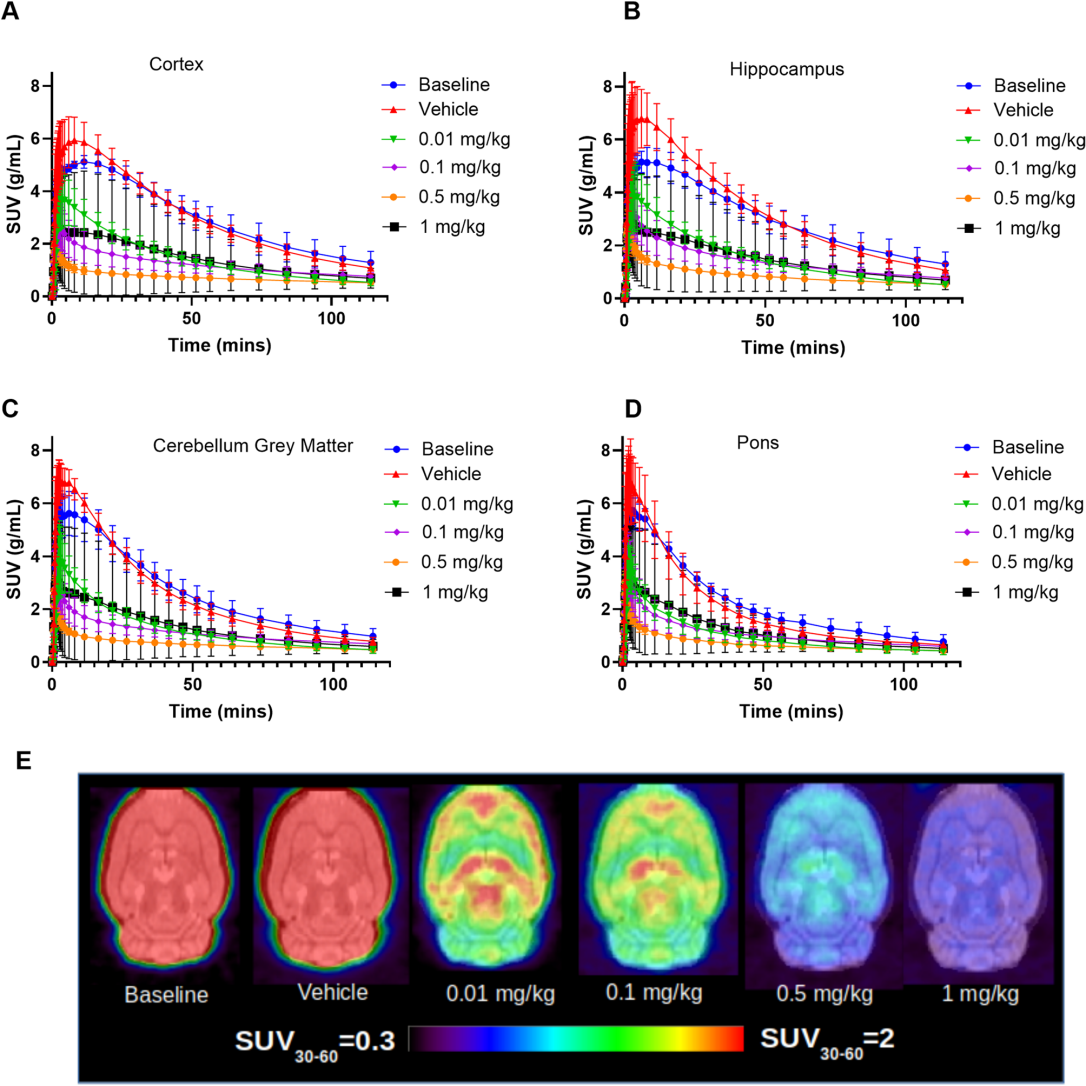
Time activity curves for four brain regions for the different injected mass levels - data presented as mean ± SD, *n* = 3 except for vehicle which is *n* = 6: cortex **(a)**, hippocampus **(b)**, cerebellum grey matter **(c)** and pons **(d)**. The TACs for the striatum and the thalamus are shown in supplementary data. Mean SUV_30 − 60__min_ images of the rat brain at 5 different injected mass levels **(e)**

**Fig. 2:**
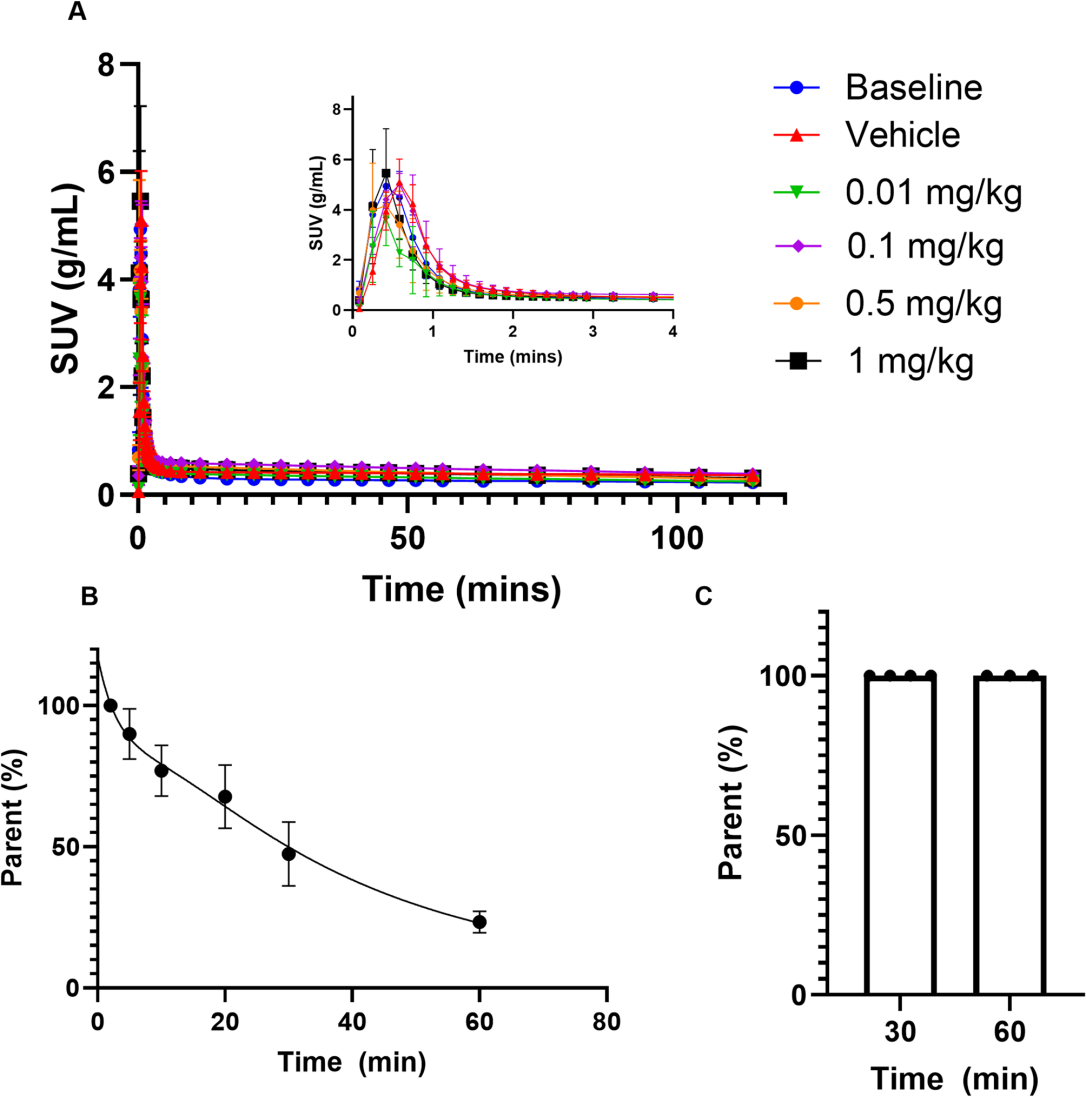
[^18^F]SynVesT-1 in whole blood as measured using the SwissTrace Twilite blood sampler for different injected mass values **(a)**. [^18^F]SynVesT-1 fraction in rat plasma **(b)** and brain tissue **(c)** over time. Data presented as mean ± SD, *n* = 3 per time point, except for brain tissue analysis at 30 min post-radiotracer injection that had *n* = 4

The time activity curves in standardized uptake value (SUV) are shown in Fig. 1 for the cortex (Fig. 1a), hippocampus (Fig. 1b), cerebellum (Fig. 1c) and the pons (Fig. 1d). The SUV curves for the striatum and the thalamus are shown in Supplementary Fig. 1. The cortex peaks at 11.5 min, the cerebellar grey matter and the hippocampus at 6 min and the pons around 3.5 min. SUV TACs for all regions show a clear blocking response between the tracer dose and the highest nonradioactive mass injected (1 mg/kg) which is also clear from the average maps of SUV presented in Fig. 1e. The arterial input function data is shown in Fig. 2a in SUV and there are no significant differences in the peak or tail of the AIFs between the doses in SUV. The radiotracer is rapidly metabolized leaving only 23.4% parent fraction in blood after 60 min (shown in Fig. 2b) but no radiometabolites enter the brain at 30 and 60 min (shown in Fig. 2c). Additionally, there is very low intra-animal variability, especially at 60 min post injection.

### [^18^F]SynVesT-1 Naïve rat brain kinetic analysis

**Fig. 3:**
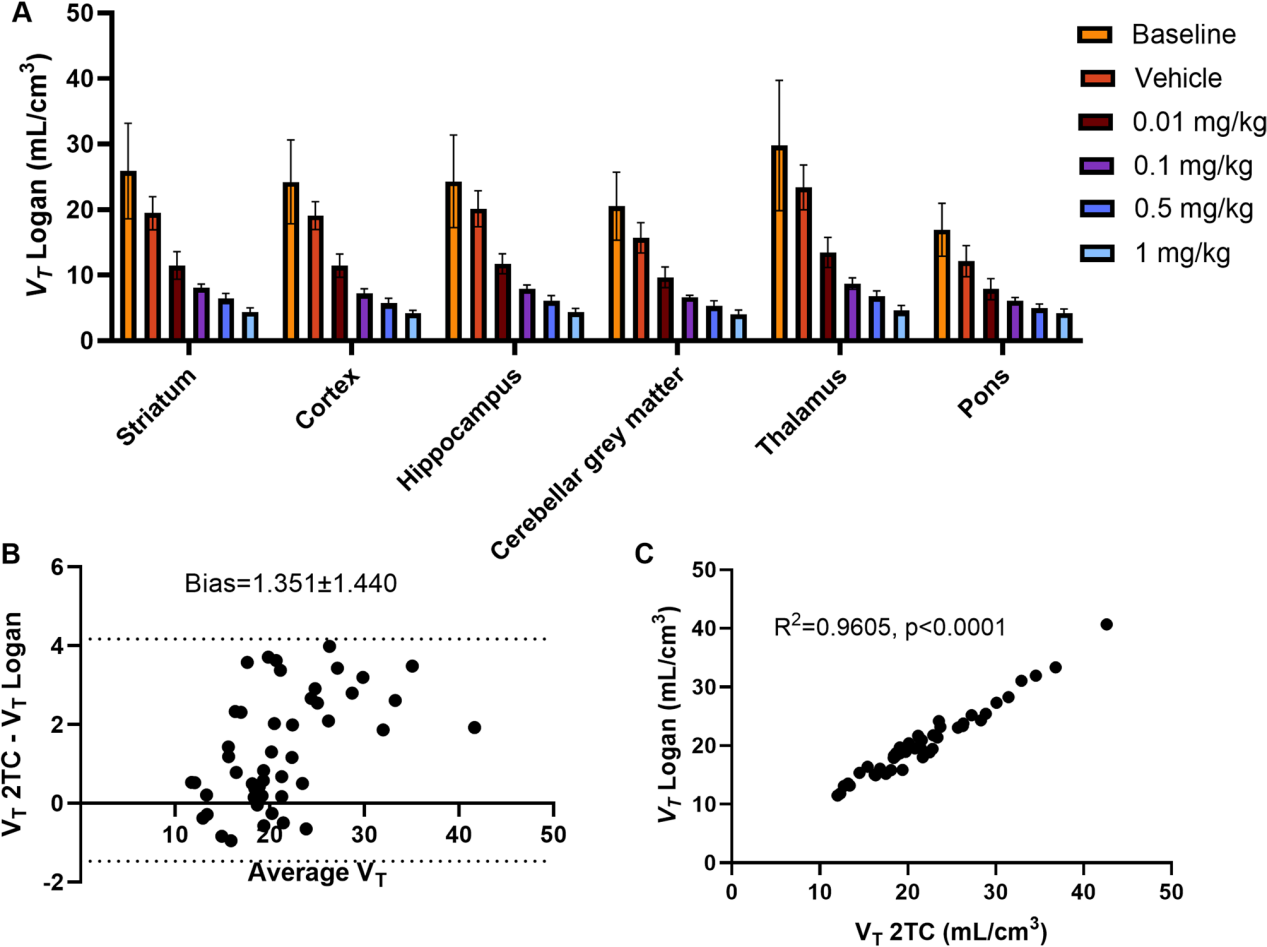
Regional *V*_*T*_ values estimated from Logan plot for different injected mass values **(a)**. Note that all the *V*_*T*_ values from the cold mass injections are significantly different at all injected mass levels for all regions compared to vehicle only– except for the pons between vehicle and the first cold dose, 0.01 mg/kg which did not reach significance. The Bland Altman plot (with 95% confidence interval bars) compares the *V*_*T*_ as estimated by the 2 tissue compartmental model and the Logan plot, and shows that there is a small absolute bias in comparison with the bias, between 4–6% depending on the region **(b)**. The correlation between the *V*_*T*_ for the 2TC and Logan plot was high and significant for the baseline scans **(c)**

**Fig. 4:**
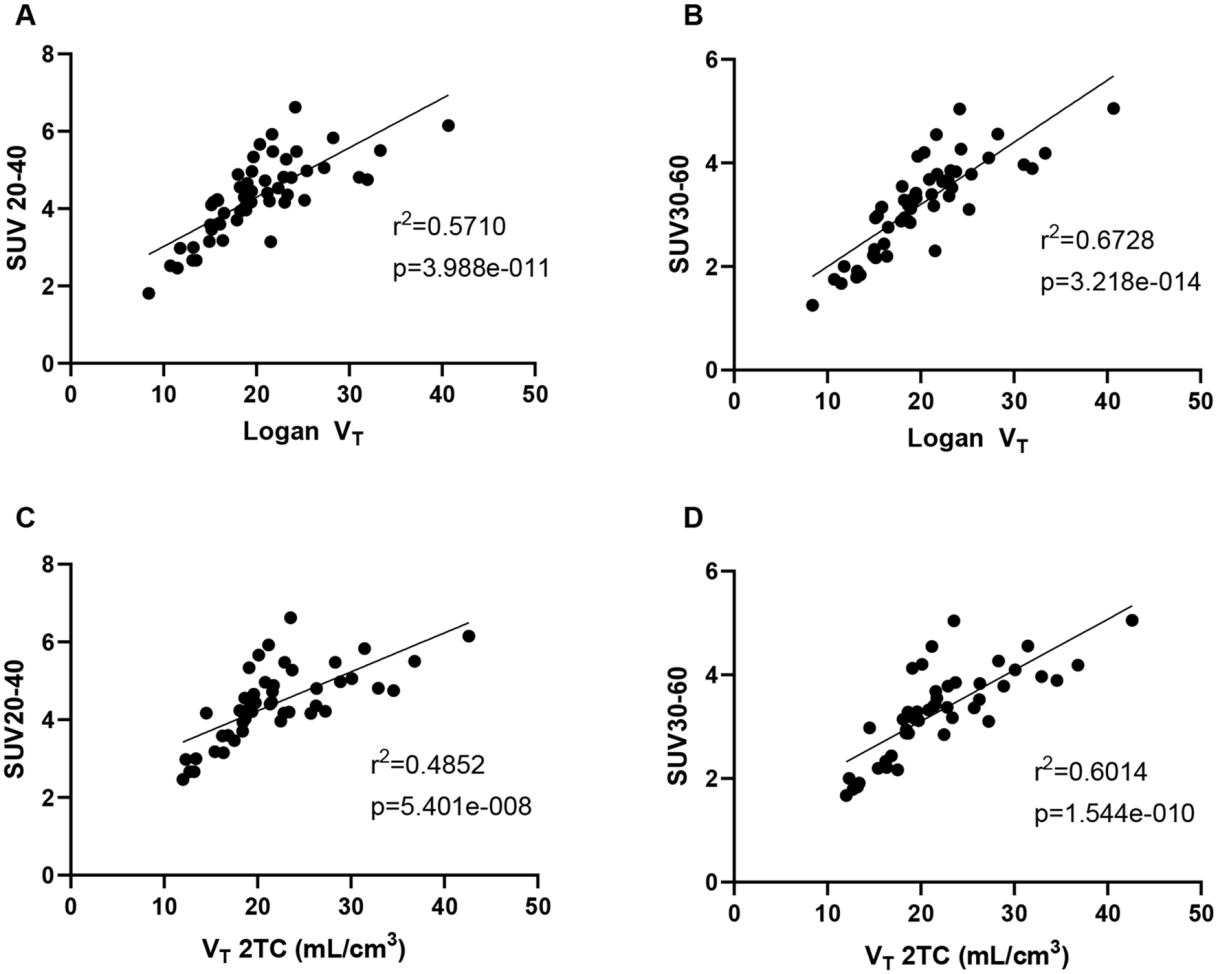
Correlations between the *V*_*T*_ and the SUV. *V*_*T*_ was estimated using the Logan plot **(a & b)** and the 2TC model **(c & d).** SUV was calculated for 20–40 min, SUV_20 − 40_**(a & c)** and 30–60 min, SUV_30 − 60_**(b & d)**

The fitting criteria (AIC) shows that the 2TC model is most appropriate for the baseline scans (Table 1), which is backed up by visual assessment of the fits. Some representative 1TC and 2TC curve fits to the PET data are shown in Supplementary Fig. 2. The Logan plot *V*_*T*_ value is highly and significantly correlated with the 2TC *V*_*T*_ (r^2^ = 0.96, *p* < 0.0001) and there is a slight bias from the Logan plot, as shown in the Bland Altman as shown in Fig. 3. The *SUV*_*20 − 40*_ and *SUV*_*30 − 60*_ are both significantly correlated with the *V*_*T*_ from 2TC modelling and Logan plot as shown in Fig. 4. The *SUV*_*30 − 60*_ is more strongly correlated with the *V*_*T*_ Logan (Fig. 4b) and *V*_*T*_ 2TC (Fig. 4d) compared to the *SUV*_*20 − 40*_ (Fig. 4a and c respectively) where the intra subject variability appears higher.

### Mass dose effect studies

**Fig. 5:**
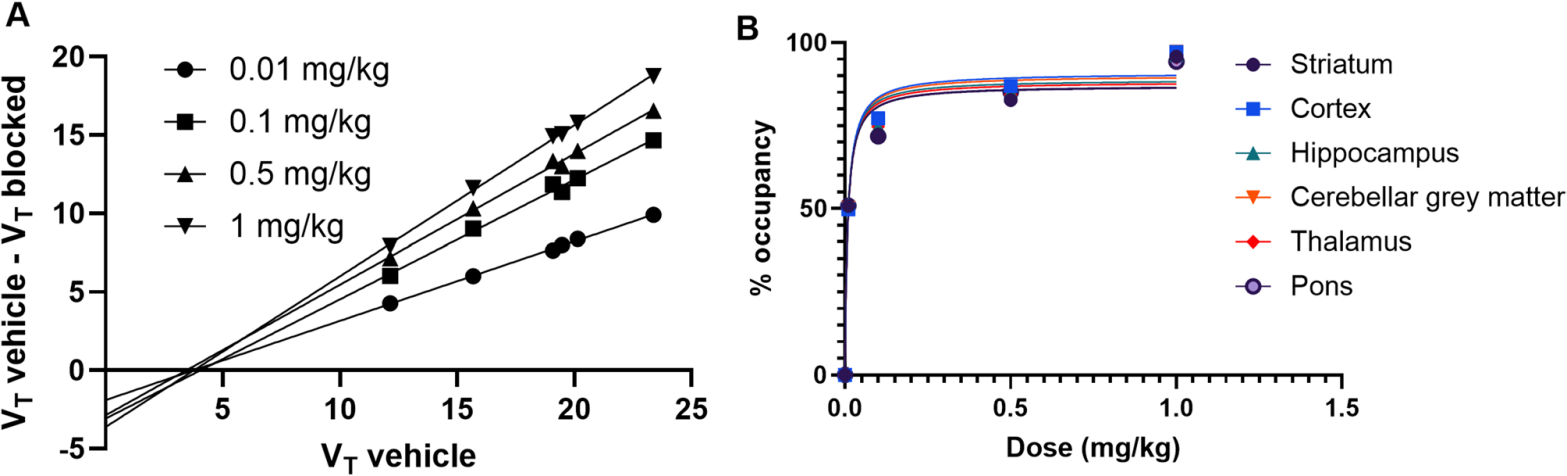
Lassen plot for [^18^F]SynvesT-1 in the rat brain **(a)**. Each point in the Lassen point represents a different brain region per injected mass level, 6 regions were used per injected dose level, with a total of 24 points. The occupancy plot **(b)** shows a similar occupancy response for all regions included

The regional *V*_*T*_ values from the Logan plot are shown in Fig. 3 for all injected mass levels. The average regional *V*_*T*_ values for the vehicle scans are between 17% and 28% lower than the baseline values depending on the region, but this difference is only significant for the striatum (*p* = 0.02), and the thalamus (*p* = 0.02). Figure 5 shows the Lassen plot which identifies a *V*_*ND*_ of 3.75 ± 0.25. The blocking is estimated at 51%, 76%, 83% and 96% for doses 0.01-1 mg/kg from the linear regression of each plot shown in Fig. 5a. These values remain similar over the regions analyzed. The occupancy plots shown in Fig. 5b were fitted with a one site specific binding curve which was deemed more appropriate than the two site binding fit due to the model being unstable and unable to estimate the 2nd binding site *B*_*max*_ and *K*_*d*_. The estimated *K*_*d*_ and upper mass dose limits from the one site specific binding fit are shown in Table 2. The upper mass dose limits for [^18^F]SynVesT-1 were 34 µg per 70 kg of body weight (average adult human) and 0.96 µg for 200 g of body weight (an average adult rat).

**Table 1:** Average *V*_*T*_ parameters and fitting criteria for each region and model tested

	2TC			1TC			Logan	
Brain region	*V*_*T*_	%SE	AIC	*V*_*T*_	%SE	AIC	*V*_*T*_	%SE
Striatum	28.12 ± 9.10	5.07 ± 3.08	43.89 ± 79.60	24.07 ± 6.36	2.23 ± 0.25	83.81 ± 82.27	25.89 ± 7.29	0.72 ± 0.15
Cortex	25.87 ± 7.25	2.23 ± 0.96	10.43 ± 92.99	22.89 ± 5.88	2.07 ± 0.09	80.34 ± 76.76	24.25 ± 6.41	0.77 ± 0.03
Hippocampus	26.23 ± 8.09	3.77 ± 1.51	48.45 ± 55.78	22.06 ± 5.89	2.86 ± 0.18	104.64 ± 74.45	24.33 ± 7.06	0.80 ± 0.09
Cerebellar grey	22.34 ± 5.53	5.19 ± 1.80	40.88 ± 77.04	18.93 ± 4.52	2.24 ± 0.25	82.43 ± 82.57	20.53 ± 5.19	0.95 ± 0.25
Thalamus	31.35 ± 10.69	5.19 ± 1.80	43.74 ± 73.86	27.36 ± 8.75	2.85 ± 0.42	104.29 ± 73.30	29.79 ± 9.94	0.74 ± 0.10
Pons	22.10 ± 13.95	37.74 ± 61.91	66.97 ± 82.36	15.05 ± 2.81	2.49 ± 0.57	86.20 ± 93.49	16.94 ± 4.06	1.03 ± 0.39

**Table 2:** Estimated equilibrium dissociation constant, upper mass dose limits *D*_*5*_ and *D*_*10*_ of [^18^F]SynVesT-1 for several brain regions of the rat

Brain region	Kd (µg/kg)	95% Confidence Interval	D5 (µg/kg)	D10 (µg/kg)
Striatum	7.88	1.062–28.73	0.48	1.02
Cortex	8.93	3.01–22.15	0.52	1.10
Hippocampus	8.07	1.86–22.15	0.48	1.02
Cerebellar grey matter	8.72	2.31–24.91	0.51	1.09
Thalamus	8.16	2.06–23.11	0.49	1.04
Pons	7.95	1.46–26.01	0.48	1.03

## Discussion

This study investigated the characterization of [^18^F]SynVesT-1 kinetics and binding parameters in the rat brain using a mass dose paradigm. This is essential for understanding how to quantify and interpret results when using [^18^F]SynVesT-1 in rat models of disease or challenge states. In plasma, the kinetic of [^18^F]SynVesT-1 is similar to that in the mouse [28] and the tracer was rapidly metabolized in vivo in blood after a bolus injection, albeit at a slower rate than for the mouse [28]. [^18^F]SynVesT-1 shows very good brain penetration and shows peak SUVs between 6 and 7 g/mL in target regions with a similar kinetic shape in line with previous studies [34]. The distribution of the radiotracer is consistent with another radiotracer of synaptic density [^11^C]UCB-J [26]. Additionally, no radiometabolites of [^18^F]SynVesT-1 were observed in the brain. The pharmacokinetics of the radiotracer are reversible and better described by the 2TC compared to the 1TC model. Logan graphical analysis shows highly and significantly correlated *V*_*T*_ values with those estimated by the 2TC and the Bland Altman plot shows good agreement between the *V*_*T*_ estimated using the two different methods with a bias of 1.35%, making it an appropriate simplification. Additionally, the *SUV*_*30 − 60* min_ shows a strong and significant correlation with the *V*_*T*_ from the Logan and 2TC model, meaning it is possible to use this metric as a surrogate for *V*_*T*_ where no blood sampling is available. The *SUV*_*20 − 40 min*_ shows significant correlation with the *V*_*T*_ from the Logan and 2TC models, however the intra-subject differences are more pronounced than in the correlation with *SUV*_*30 − 60 min*_, which also has a higher r^2^ value.

The radiotracer binding was blocked in all brain regions in a dose-dependent manner by non-radioactive SynVesT-1, with a large reduction in SUV, between 68% (pons) and 81% (thalamus) for the highest cold injected mass of 96% global occupancy. There was no brain region that didn’t show a response to the increasing cold mass injection, indicating that there is no brain region without target binding, thus no region that can be used as a true reference region. The region with the lowest binding of [^18^F]SynVesT-1 was shown to be the pons but it still has significant amounts of SV2A present and showed a strong response to increasing mass dose. This is in line with previous studies in the mouse [28], which show a similar blocking pattern over all brain regions, and comparable *V*_*T*_ values, with the same lack of true reference region. Several studies have used reference regions in rodents—such as white matter [35], brain stem [8], cerebellum [9, 36], striatum [37] —to quantify [^18^F]SynVesT-1 binding to SV2A. However, caution is advised, as any changes in SV2A levels within the reference region can affect binding parameters in target regions. These reference regions should be considered pseudo-reference regions and used only when the chosen area has been validated to be free from pathology or age-related changes for the specific species being studied.

The Lassen plot revealed a *V*_*ND*_ of 3.75, which is relatively low compared to the peak *V*_*T*_ value in the thalamus showing a contribution of 12.5%, and 22% for the pons which has the lowest *V*_*T*_ value. This means that the *V*_*T*_ value will be reflective of the SV2A binding more than changes to tracer perfusion into tissue or non-specific binding.

The upper mass dose limit for [^18^F]SynVesT-1 is small for human studies (shown as 34 µg per 70 kg of body weight with a 5% receptor occupancy, extrapolated from the rat), requiring a sufficiently high molar activity to balance the injected radioactivity dose and the injected mass for a tracer dose experiment thereby avoiding any mass dose effects. The injected activity is either 180 MBq [16] or 3.7 MBq/kg (259 MBq for 70 kg human) [18] which requires a molar activity of 1.6 GBq/µmol or 2.3 GBq/µmol respectively to achieve the upper mass limit– this is achievable and exceeded in most recent published studies [38, 39] as well as the present study (molar activity presented in Methods). For a rat with average weight of 0.48 kg and an injected dose of ~ 37 MBq, the required molar activity is 48.5 GBq/µmol in order to achieve less than 5% occupancy (upper mass limit of 0.48 µg/kg) and 22.8 GBq/µmol to achieve less than 10% occupancy (upper mass limit of 1.02 µg/kg).

The molar activity at end of synthesis for this study (shown in the Methods) achieved this minimum required, except for the initial baseline experiments. The low molar activity resulted in an occupancy of between 16 and 33%, as based on the occupancy curve from the mass dose experiment, highlighting the importance of achieving a sufficiently high molar activity– especially if scanning will not take place directly post-synthesis or when imaging small animals. The three baseline scans were used to validate the kinetic model choice and the use of the Logan plot and *SUV*_30–60_ and were not incorporated into the analysis of the mass dose experiments. Interestingly, despite the self-blocking, the regional *V*_*T*_ values of the baseline group remained higher than those found for the vehicle group– only significant for the thalamus and the cortex– thus, highlighting the effect of vehicle on radiotracer brain kinetics. This is likely a result of vehicle effects on radiotracer uptake kinetics, as different vehicle solutions are known to impact animals physiology differently [40, 41] thereby impacting pharmacokinetic and pharmacodynamic of compounds in vivo.

Results in this study confirm data recently published with [^18^F]SynVesT-1 in the rat [29] while expanding remit to provide full occupancy plot and *V*_*ND*_ estimates. The present study also demonstrates the importance of consideration of the effect of vehicle solutions on radiotracer kinetics in vivo. In contrast with the recent report carried out exclusively in female rats [29] our study used male rats only. The differences in reported *V*_*T*_ values in our study versus the Berckmans et al. study may be due to sex-differences, or scanner differences, although separate single sex studies are limited with regards to kinetic assessments generalizable to both sexes.

## Conclusion

[^18^F]SynVesT-1 displayed excellent brain penetration in the rat and the distribution is consistent with other SV2A tracers in the rat and mouse. The data show that there is no true reference region available for simplified quantification, however the *SUV*_*30 − 60 min*_ could be used as a proxy for *V*_*T*_ when blood sampling is not available as it shows a correlated, albeit biased value. The *V*_*ND*_ is relatively low compared to the regional *V*_*T*_ values, which means that the *V*_*T*_ will be highly reflective of changes in tracer binding to SV2A protein. The mass dose upper limit for occupancy < 5% in humans is 34 µg per 70 kg of body weight (extrapolated from the rat), requiring careful monitoring of molar activity to avoid any mass effects in experiments. This is especially relevant for pathologies where a reduction in SV2A is expected to be low. Overall, [^18^F]SynVesT-1 shows great potential as an imaging biomarker for neurological and neurodevelopmental disorders in the rat.

## Electronic supplementary material

Below is the link to the electronic supplementary material.

Supplementary Material 1

## Data Availability

The datasets generated and analyzed during the current will be made available on the University of Edinburgh Datashare website (https://datashare.ed.ac.uk/), or will be made available from the corresponding author upon reasonable request.
