# Supplementary material for: Characterization of in vivo binding kinetics and non-displaceable binding of [18F]SynvesT-1 in the rat brain
Source: EJNMMI Res. 2025 Jun 18;15:72. doi: 10.1186/s13550-025-01270-2 (PMC12176722; doi:10.1186/s13550-025-01270-2)
Supplement: Supplementary file 1 — Supplementary Material 1 [file 13550_2025_1270_MOESM1_ESM.docx]

Supplementary materials:

Supplementary Figure 1: Time activity curves for striatum (a) and thalamus (b) at the different dose levels.

Supplementary Figure 2: 1TC and 2TC fits of a cortex TAC from one representative baseline scan
